# Efficacy and Safety of Third Dose of the COVID-19 Vaccine among Solid Organ Transplant Recipients: A Systemic Review and Meta-Analysis

**DOI:** 10.3390/vaccines10010095

**Published:** 2022-01-09

**Authors:** Orly Efros, Roi Anteby, Mirit Halfon, Eshcar Meisel, Eyal Klang, Shelly Soffer

**Affiliations:** 1 National Hemophilia Center, Thrombosis & Hemostasis Institute, Sheba Medical Center, Ramat-Gan 5262100, Israel; 2 Sackler Faculty of Medicine, Tel-Aviv University, Tel-Aviv 6997801, Israel; Roi.Anteby@sheba.gov.il (R.A.); mirithalfon@mail.tau.ac.il (M.H.); eshcarme@gmail.com (E.M.); Eyal.Klang@sheba.gov.il (E.K.); 3 Department of Surgery and Transplantation B, Sheba Medical Center, Ramat-Gan 5262100, Israel; 4 Internal Medicine Department D, Sheba Medical Center, Ramat-Gan 5262100, Israel; 5 Department of Diagnostic Imaging, Sheba Medical Center, Ramat-Gan 5262100, Israel; 6 Sheba Talpiot Medical Leadership Program, Ramat-Gan 5262100, Israel; 7 Internal Medicine Department B, Assuta Medical Center, Ben-Gurion University of the Negev, Be’er Sheva 8410501, Israel; soffer.shelly@gmail.com

**Keywords:** COVID-19, vaccines, third dose, solid organ transplant recipients

## Abstract

Solid organ transplant recipients were demonstrated to have reduced antibody response to the first and second doses of the COVID-19 mRNA vaccine. This review evaluated published data on the efficacy and safety of the third dose among solid organ transplant recipients. We performed a systematic search of PubMed, EMBASE, and Web of Science to retrieve studies evaluating the efficacy of the third dose of anti-SARS-CoV-2 vaccines in adult solid organ transplant recipients. Serologic response after the third vaccine was pooled using inverse variance and generalized linear mixed and random-effects models. Seven studies met our inclusion criteria. A total of 853 patients received the third dose. Except for one randomized controlled trial, all studies were retrospective in design. Following the third COVID-19 vaccine dose, antibody response occurred in 6.4–69.2% of patients. The pooled proportion of antibody response rate after the third vaccine was 50.3% (95% confidence interval (CI): 37.1–63.5, I^2^ = 90%). Five papers reported the safety profile. No severe adverse events were observed after the third vaccine dose. In conclusion, a third dose of the SARS-CoV-2 mRNA vaccine in solid organ transplant recipients is associated with improved immunogenicity and appears to be safe. Nevertheless, a significant portion of patients remain seronegative.

## 1. Background

The ongoing coronavirus disease 2019 (COVID-19) pandemic caused by SARS-CoV-2 has affected billions of lives throughout the world. Solid organ transplant recipients are at a greater risk of severe disease and death following COVID-19. One study reported a mortality rate of more than 20% among solid organ recipients hospitalized with COVID-19 [1]. The increased risk is mainly attributed to the patients’ higher burden of comorbidities and older age rather than immunosuppression intensity-related measures [1,2].

Efforts to overcome the spread of COVID-19 have focused on the development and distribution of anti-SARS-CoV-2 vaccines. The efficacy and safety of mRNA COVID-19 vaccines have been well demonstrated in both large-scale phase III trials and real-world data. The high effectiveness of the mRNA-based vaccines shown by these studies has led to mass vaccination campaigns against COVID-19 [3,4,5,6]. However, solid organ recipients were excluded from phase III trials; therefore, there is no evidence to show the efficacy and safety of the vaccines in this immunocompromised population. Recent reports show a reduced antibody response among solid organ transplant recipients after the first and second mRNA vaccine doses [7,8,9,10,11]. Boyarsky et al. reported that most solid organ transplant recipients did not respond with an appreciable anti-spike antibody level following the first dose [8]. A successive study by Boyarsky et al. demonstrated that most solid organ transplant recipients had a detectable antibody response after the second dose. However, the antibody levels measured were below the observed levels in immunocompetent vaccinated individuals [7]. Grupper et al. analyzed the humoral response in 136 kidney transplant recipients and compared it to 25 controls following vaccination with the BNT162b2 (Pfizer/BioNTech). A positive antibody response developed in only 51 of 136 transplant recipients (37.5%), while 100% of controls had positive serology [9]. The rate of side effects was similar between these groups [9].

The disappointing immunologic response of solid organ transplant recipients following two vaccine doses led to the authorization of a third dose in several countries, such as France, Israel, and Germany [12,13,14,15,16,17,18].

This review evaluated published data on the efficacy and safety of the third dose among solid organ transplant recipients.

## 2. Patients and Methods

### 2.1. Study Design

A systematic review and a meta-analysis were conducted according to the Preferred Reporting Items for Systematic Reviews and Meta-Analysis guidelines (PRISMA) [19]. Before commencing the study, the protocol was registered with PROSPERO, a prospective international register of systematic reviews (CRD42021284154).

### 2.2. Inclusion Criteria and Search Strategy

Studies evaluating the efficacy of a third dose of anti-SARS-CoV-2 vaccines in solid organ transplant recipients over 18 years of age were included. Studies of patients with previous COVID-19 infection or positive serology prior to first vaccination were excluded.

A systematic literature search was performed using PubMed, EMBASE, and Web of Science on 26 September 2021. Search keywords included (“three doses” or “third dose” or “booster” or “third-dose” or “third COVID-19 vaccine” or “3rd dose” or “3 doses” or “prime-boost vaccination” or “three-dose” or “third doses” or “third shot” or “third shots”) AND (“solid-organ transplant recipients” or “immunosuppressed” or “transplant recipients” or “organ-transplant recipients”) AND (“COVID-19 Vaccine” or “messenger RNA vaccine” or “mRNA vaccine” or “Pfizer” or “mRNA-1273” or “BNT162b2” or “COVID-19 mRNA vaccine” or “SARS-CoV-2 Vaccine” or “BioNTech” or “mRNA vaccination” or “messenger RNA-based vaccines” or “anti-SARS-CoV-2”). The complete search strategies can be found in Supplementary Document S1. Randomized controlled trials (RCTs) and observational studies were included, while case reports, reviews, and expert opinions were excluded. Only English language papers were included.

### 2.3. Study Selection

Two authors (S.S. and R.A.) independently screened each article title and abstract for inclusion and exclusion criteria. The full-text article was reviewed when meeting the inclusion criteria or encountering any uncertainty. A third reviewer (O.E.) resolved disagreements. Finally, references of included articles were further screened to identify additional relevant studies. For serial studies, we included the most updated publication to avoid repeated inclusion of the same patients.

### 2.4. Data Extraction

Two reviewers (S.S. and R.A.) extracted data using a standardized data extraction sheet. Data included the following information: first author, date of publication, study design, study period, data source, study location, the total number of participants, demographic information including age and sex, inclusion and exclusion criteria, the status of previous COVID-19 diagnosis, the type of vaccine used, the time between second and third vaccine dose, serologic response before the third vaccine, type of organ transplant, time from transplantation, immunosuppression maintenance therapy, percentage of patients with serological response to the third vaccine dose, type of tests used for outcome evaluation, the time between the third dose to outcome evaluation, and other relevant outcomes when available. Statistical analysis methods and adjustment for confounders were noted.

### 2.5. Outcomes

The primary outcome was vaccine effectiveness, defined as a serologic response after the third vaccine. The response was determined for each study according to the manufacturer thresholds. Seroprevalence was defined in each original study via a threshold of SARS-CoV-2-specific antibodies, according to specific manufacturer recommendations.

### 2.6. Assessment of Study Quality

The evidence quality of included studies was assessed according to the Grading of Recommendations, Assessment, Development, and Evaluation (GRADE) criteria [20]. GRADE expresses the degree of confidence in the quality of evidence and strength of recommendation [20]. In addition, the Risk of Bias in Nonrandomized Studies of Interventions (ROBINS-I) tool was used to assess the risk of bias [21]. Two authors (S.S. and R.A.) independently evaluated each study using the GRADE and ROBINS-I framework, and non-consensus was resolved by a third author (OE).

### 2.7. Statistical Analysis

A random-effects meta-analysis synthesized a pooled proportion with a 95% CI. A logit transformation was performed as suggested by Warton and Hui [22]. The pooled results were based on the inverse variance method, with a sensitivity analysis conducted using the generalized linear mixed models (GLMM), which was reported to be superior in the analysis of small sample sizes [23]. For the inverse-probability method, the DerSimonian–Laird estimator was used to estimate the between-study variance. For the GLMM methods, the maximum-likelihood estimator was utilized. Heterogeneity between studies was defined as low (I^2^ = 0–25%), moderate (I^2^ = 26–75%), and high (I^2^ > 75%). Funnel plots and the Egger test were considered for the assessment of publication bias. Statistical analysis was conducted using the metaprop package in R 4.1.1 software (Comprehensive R Archive Network, http://cran.r-project.org/ (accessed on 30 October 2021)).

## 3. Results

### 3.1. Bibliographic Search and Study Selection

**Figure 1:**
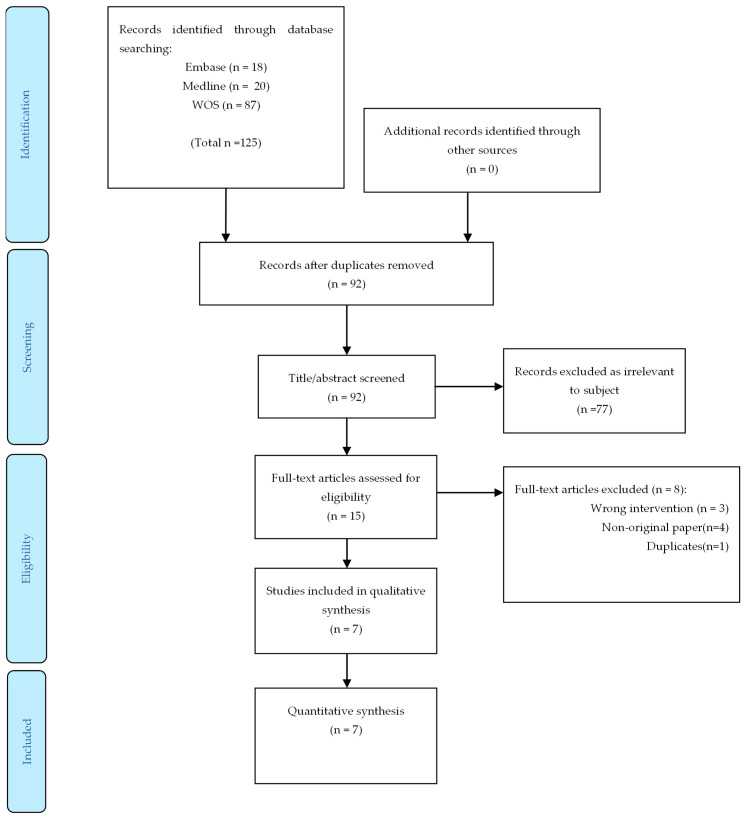
Flow diagram of retrieval of studies from the literature review. WOS, Web of Science.

The literature search identified 125 potential publications. After excluding duplicates and ineligible studies by title or abstract screening, 15 studies underwent full-text review. A final seven studies were included in the quantitative meta-analysis (Figure 1). We did not include the study by Kamar et al. [24] since the same data were used in a subsequent report by Del Bello et al. [24].

### 3.2. Characteristics of the Included Studies

Details of included studies (*n* = 7) are presented in Table 1. All seven studies were published in the second half of 2021. Most (*n* = 4, 57%) were conducted in France [13,15,16,25]. One study by Hall et al. was a randomized control trial study [18]. The other studies were retrospective single-arm cohort studies in design. The distribution between the mRNA-1273 vaccine (Moderna) (*n* = 3, 42%) and mRNA-BNT162b2 vaccine (Pfizer–BioNTech) (*n* = 3, 42%) was balanced. In one study, three vaccines were used: 50% of the participants received the Ad26.COV2.S vaccine (J&J/Janssen), 30% received the mRNA-1273 vaccine, and 20% received the 162b2 vaccine [14]. Between the second dose and the third dose of vaccine administration, the time ranged from 50 to 70 days.

### 3.3. Patient Characteristics

A total of 913 patients were included in the final review. Of them, 853 patients received the third dose, and 60 patients received a placebo as part of a randomized controlled trial by Hall et al. [18]. The largest studies were by Del Bello et al. and Benotmane et al., comprising 396 and 159 patients, respectively. Across all studies, 43% to 80% of patients were male. Four studies evaluated patients exclusively after kidney transplant, while three evaluated patients after kidney or other organ transplants, including lung, heart, liver, and pancreas. All studies reported the rate of seropositivity following COVID-19 vaccination as a primary outcome. The prevalence of patients with a serologic response before receiving the third vaccine dose varied between 0% and 49.7%. The median time from transplantation to the third vaccine was between 3.16 and 10.5 years. All reports included patients concomitantly treated with calcineurin inhibitor treatment and steroid treatment. One study focused on patients treated with belatacept.

### 3.4. Primary Endpoint Measurement

In four reports (57%), the serology response measurements were performed using an electrochemiluminescence anti-SARS-CoV-2 immunoassay (Roche Elecsys^®^ (Roche, Mannheim, Germany and Roche Diagnostics, Basel, Switzerland ). In three reports (*n* = 3, 42%), measurements were performed using the chemiluminescent microparticle immunoassay (Abbott Architect^®^ (Abbott Laboratories, Illinois, United States) (Table 2). The time between the third dose vaccine administration and the serology test ranged from 2 to 4 weeks.

### 3.5. Safety

Five papers reported the safety profile of the third vaccine dose [13,14,15,18,25]. In all studies, no severe adverse events were noted (Table 2).

### 3.6. Quality of Evidence and Risk of Bias Assessments

The quality of evidence assessment and risk of bias of included studies are summarized in Table 3 and Supplementary Table S1. According to the GRADE criteria, the quality of evidence was “low” in three studies [14,17,25], “moderate” in three study [13,15,16], and “high” in one study [18]. According to the ROBINS-I tool, the risk of bias was “moderate” in five studies [14,15,16,17,25] and “serious” in one study [13]. The article by Hall et al. was not evaluated by ROBINS-I as this tool is not applicable for randomized control trials.

### 3.7. Antibody Response Following a Third Dose

**Figure 2:**
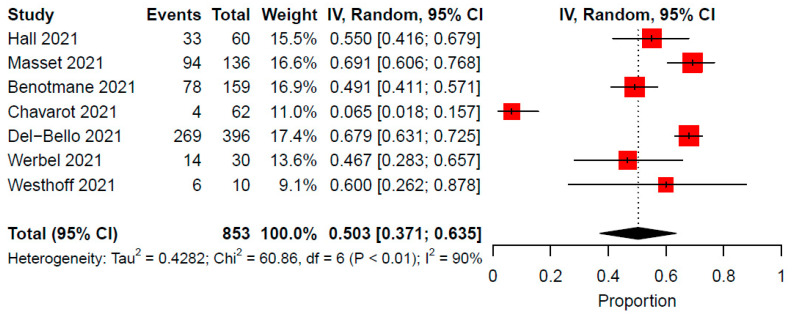
Proportion of antibody response after the third COVID-19 vaccine dose among solid organ transplant recipients. CI, confidence interval; IV, inverse variance.

Antibody response following the third COVID-19 vaccine dose occurred in 6.4–69.2% of patients across included studies. In a meta-analysis of seven studies comprising of 853 immunocompromised patients, the pooled rate of antibody response after the third vaccine was 50.3% (95% CI: 37.1–63.5, I^2^ = 90%) (Figure 2). A sensitivity analysis using the GLLM produced similar proportion estimates (Supplementary Figure S1). In the GLLM meta-analysis (studies = 7, patients = 853), the pooled rate of antibody response following a third dose of the vaccine was 48.4% (95% CI: 29.9-67.4, I^2^ = 90%). Publication bias assessment using funnel plots and Egger tests was not feasible due to the small number of studies included in the pooled analyses.

### 3.8. Seroconversion Rate between the Second and Third Vaccine Doses

**Figure 3:**
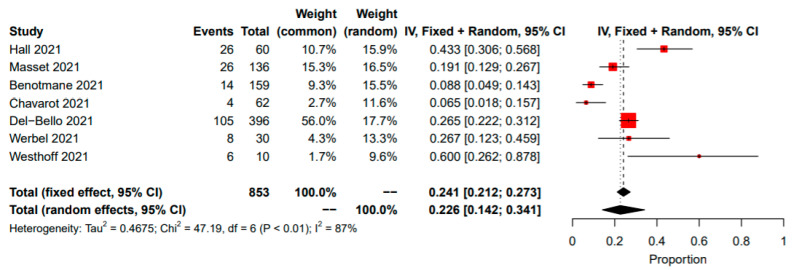
Proportion of seroconversion between the second and third doses of COVID-19 vaccine in solid organ transplant recipients. CI, confidence interval; IV, inverse variance.

The pooled proportion of patients that were seronegative after the second dose and transitioned to seropositivity after the third dose was 22.6% (95% CI: 14–34%; I^2^ = 87%, *p* < 0.01) (Figure 3).

## 4. Discussion

Recent studies showed a substantially decreased antibody response of solid organ transplant recipients to the first two doses of the SARS-CoV-2 mRNA [7,8,9,10]. A second vaccine dose was demonstrated to have an improved response in anti-spike antibodies in transplant recipients compared with the first dose [7]. However, it was regarded as suboptimal, raising hope for an additional benefit of a third dose in this population. France, Israel, and Germany were some of the first countries to authorize solid organ transplant recipients to receive a third dose of the SARS-CoV-2 mRNA vaccine [12]. Nevertheless, some patients were reported to receive the third dose of their own accord in other countries [26]. With the accumulating data regarding the potential benefit of a third dose, in August 2021, the United States Food and Drug Administration (FDA) issued an emergency use authorization for the third dose of the mRNA-based vaccine for immunocompromised patients, including solid organ transplant recipients [27].

This meta-analysis showed that solid organ transplant recipients had an improved immunogenic response to the third dose of the COVID-19 vaccine. Nevertheless, this response appears to be reduced when compared to the reported response of the average population. No severe or life-threatening events were reported among transplant recipients after receiving the third vaccine dose.

Seven studies were included in this review. In a randomized controlled trial by Hall et al., 120 organ-transplant recipients received the third dose of the mRNA-1273 (Moderna) vaccine or a placebo [18]. Positive antibody response was significantly more prevalent in the mRNA-1273 group compared to the placebo (55% versus 18% respectively). In a large retrospective study from France of 396 solid organ transplant recipients, after receiving the third dose, the positive anti-SARS-CoV-2 antibody prevalence increased from 41.4% to 67.9% [13]. In addition, 45.3% of the seronegative patients turned positive after the third dose. Other retrospective studies affirmed these results with increased serologic response rates after the third dose [14,15,16,17].

Less encouraging results were demonstrated in a study conducted by Chavarot et al., which evaluated the response of 64 seronegative kidney transplant recipients treated with belatacept [25]. The seropositivity rate did not improve significantly after receiving the third dose of BNT162b2-mRNA (Pfizer/BioNTech) vaccine, with only 6.4% developing low anti-SARS-CoV-2 antibody titers following the third dose.

Factors associated with reduced serologic response and lower seroconversion rates after the third mRNA vaccine comprised older age, a lymphocyte count lower than 1500/mm^3^, impairment of the allograft function, and treatment with mycophenolic acid, belatacept, or at least a triple immunosuppression regime [13,15,16]. One study indicated that male recipients were more likely to respond [16]. The other studies did not demonstrate an association between gender and vaccine response. Two of the included studies evaluated the cellular response following the third vaccine dose. Hall et al. showed a greater increase in SARS-CoV-2–specific T-cell counts after the third mRNA-1273 vaccine dose when compared to placebo [18]. Another study showed a substantial increase in SARS-CoV-2 spike protein-reactive T-cell immunity in 90% of the patients receiving the third dose, as well as increased frequencies of cytokine-producing T cells and follicular T helper cells. This suggests improved antiviral functionality [17].

The results of the studies included in this meta-analysis were coherent. Most studies demonstrated a substantial benefit of the third dose of SARS-CoV-2 mRNA vaccine in solid organ transplant recipients. An exception to this finding was described in a study that included only belatacept-treated patients. We also showed that a significant proportion of seroconversion followed the third vaccine dose.

The safety profile assessed in the various studies showed consistent results. No anaphylactoid reactions were observed, and no life-threatening events were noted [13,14,17,18,25]. Common adverse events included mild to moderate local reactions and mild or moderate fatigue. Reactions such as severe arm pain, severe headache, and severe myalgia were also noted [14]. One heart transplant recipient had an acute volume overload 7 days after her third dose, representing biopsy-proven antibody-mediated rejection. However, her heart function was preserved, and she did not require immunosuppressive intensification. Moreover, the patient’s COVID-19 antibody titer profile was not increased, and no clear link to the third dose vaccine can be made [14].

This meta-analysis had some limitations. First, the small cohort included in each study reduced its statistical power. Second, there was heterogeneity between studies regarding the vaccine type, timing of serological tests before and after the third dose, and definition of antibody positivity. It should be noted that there is currently no universal defined cutoff value for a positive antibody response. In addition, the role of the serologic response in the immunity and protection from COVID-19 has not been fully established, and recent studies have suggested that T-cell response may have a higher contribution to controlling severe COVID-19 [28,29]. Nevertheless, some studies evaluated the cellular response after the third dose and showed similar results. Lastly, the short follow-up and the lack of COVID-19 incidence rates following the third dose limit our conclusions.

## 5. Conclusions

In solid organ transplant recipients, a third dose of the SARS-CoV-2 mRNA vaccine is associated with improved immunogenicity and appears to be safe. Nevertheless, a significant proportion of solid organ transplant recipients remain seronegative after receiving the third vaccine dose.

## Figures and Tables

**Table 1:** Characteristics of included studies.

Author (Month Year)	Country	Design	Participants	No. of Participants	Males (%)	Age Median/Mean (Range/SD)	Organ Transplanted	Immunosuppression	Time from Transplantation (Median, IQR)	Third Vaccine Dose	Time from 2nd to 3rd Dose	Antibody Titer before 3rd Dose
Hall (Sep 2021)	Canada	RCT	Solid-organ transplant recipients	120	61.7%	66.6 (63.3–71.4)	Lung (11%), heart (10%), kidney (20%), pancreas, kidney–pancreas (15%), liver (4%)	Steroids (83.3%), calcineurin inhibitor (98.3%), MMF/mycophenolate sodium (73.3%), azathioprine (13.3%), sirolimus (10%)	3.16 (1.71–6.12) years	mRNA-1273 (Moderna)	Two months	11.70%
Masset (Aug 2021)	France	Retrospective	Kidney transplant recipients	136	63.20%	63.7 (±11.7)	Kidney (100%)	Calcineurin inhibitor (85.8%), mTOR inhibitor (14.9%), antimetabolite (75.4%), steroids (32.1%)	10.5 (±8.5) years	mRNA-BNT162b2 (Pfizer/BioNTech)	50 days	49.70%
Benotmane (Jul 2021)	France	Retrospective	Kidney transplant recipients with minimal serologic response to two doses	159	61.60%	57.6 (49.6-66.1)	Kidney (100%)	Tacrolimus + MMF/MPA + steroids (52.8%)	5.3 (1.9–11.1) years	mRNA-1273 (Moderna)	51 days (IQR, 48–59 days)	40.3% (0 patients were considered positive)
Chavarot (Aug 2021)	France	Retrospective	Belatacept-treated kidney transplant recipients	62	58%	63.5 (51–72)	Kidney (100%)	Belatacept + MPA (71%), mTOR inhibitor (13%), azathioprine (5%), steroids (100%)	47.5 (25.3–79) months	mRNA-BNT162b2 (Pfizer/BioNTech)	69.5 days (IQR 40-84)	0.00%
Del Bello (Jul 2021)	France	Retrospective	Solid organ transplant recipients	396	65%	59 ± 15	Kidney (69.9%), liver (14.2%), heart or lung (8.5%), pancreas (1.5%), combined (2.5%)	Calcineurin inhibitor (86.1%), MPA (71.9%) mTOR inhibitor (26.7%), belatacept (6.3%), steroids (82.0%)		mRNA-BNT162b2 (Pfizer/BioNTech)	59 days (IQR 47–67)	41.4% (95% CI, 36.5% to 46.3%
Werbel (Sep 2021)	USA	Retrospective	Solid organ transplant recipients	30	43%	57 (44–62)	Kidney (73.3%), liver (10%), heart (6.6%), pancreas (3.3%), lung (3.3%), combined (3.3%)	Tacrolimus or cyclosporine + MPA (83.3%), steroids (80%), sirolimus (3.3%), belatacept (3.3%)	4.5 (2.3–10.5) years	Ad26.COV2. S (50%)(J&J/Janssen), mRNA-1273 (30%) (Moderna), 162b2 (20%) (Pfizer/BioNTech)	67 days (IQR, 54 to 81)	20%
Westhoff (Sep 2021)	Germany	Retrospective	Kidney transplant recipients	10	80%	60 (51.5–67.5)	Kidney (100%)	Calcineurin inhibitor (80.0%), MPA (90%), mTOR inhibitor (10.0%), belatacept (10.0%), steroids (100.0%)	41.5 (21.75–89.25) months	mRNA-1273 (Moderna)	10 (10–10) weeks	0

No.—number; SD—standard deviation; IQR—interquartile range; RCT—randomized controlled trial, MMF/MPA—mycophenolic acid; mTOR—mammalian target of rapamycin.

**Table 2:** Efficacy and safety of the third dose of COVID-19 vaccine among solid organ transplant recipients.

Author (Month Year)	Primary Outcome	Time from 3rd Vaccine to Outcome Measuring	Primary Outcome Measuring Technique	Seropositive Rate (%)	Secondary Outcome	Secondary Outcome Results	Factors Associated with Reduced Response	Safety
Hall (Sep 2021)	Serologic response	One month	Electrochemiluminescence immunoassay (Roche Elecsys^®^)	55%	1. The median percent virus neutralization 2. The polyfunctional T-cell response	1. 71% 2. The median SARS-CoV-2–specific T-cell counts were greater, with a minimal polyfunctional CD8^+^ T-cell response		Local and systemic events were slightly more common after the third dose of mRNA-1273. No grade 3 or 4 events and no cases of acute rejection occurred.
Masset (Aug 2021)	Serologic response	One month	Chemiluminescent microparticle immunoassay (Abbott Architect^®^) or chemiluminescence immunoassay (Siemens Atellica^®^), or electrochemiluminescence immunoassay (Roche Elecsys^®^)	69.2%	Antibody titer concentration	Low antibody titers (median 209, IQR (20-409) AU/mL)	1. Lymphocyte count < 1500/mm^3^ 2. Impairment of allograft function 3. Female recipients	
Benotmane (Jul 2021)	Serologic response	28 days (IQR, 27–33)	Chemiluminescent microparticle immunoassay (Abbott Architect^®^)	49%			1. Patients without an antibody response 2. Patients receiving tacrolimus, mycophenolate, and steroids	No severe adverse events were observed after the third dose.
Chavarot (Aug 2021)	Serologic response	28 days (IQR, 28-33)	Chemiluminescent microparticle immunoassay (Abbott Architect^®^)	6.4%				No patient presented severe systemic events.
Del Bello (Jul 2021)	Serologic response	Four weeks	Enzyme-linked immunosorbent assay (Beijing Wantai Biological Pharmacy Enterprise)	67.9%			1. Older patients 2. Patients receiving mycophenolic acid, belatacept 3. Patients receiving at least a triple immunosuppression	No serious adverse event or acute rejection episode was observed.
Werbel (Sep 2021)	Serologic response	14 days (IQR, 14–17)	Electrochemiluminescence immunoassay (Roche Elecsys^®^ or Quantitative ELISA (EUROIMMUN)	46.6%				No patient presented severe systemic events. One heart transplant recipient had a biopsy-proven, antibody- mediated rejection seven days after the third dose in the setting of acute volume overload.
Westhoff (Sep 2021)	Serologic response	Two weeks	Electrochemiluminescence immunoassay (Roche Elecsys^®^)	60%	1. Antibody titer concentration 2. Reactive T cells response	1. Median antibody titer concentration of 542 (IQR, 478–923) U/mL 2. A substantial increase in the magnitude of SARS-CoV-2 spike (S) protein–reactive T-cell immunity in 90% of subjects		

**Table 3:** Quality assessments of included studies.

Author (Month Year)	Certainty of the Evidence (GRADE) ^20^ *	Risk of Bias Assessment (ROBINS-I) ^21 #^
Hall (Sep. 2021)	High	N/A
Masset (Aug. 2021)	Moderate Due to the risk of bias	Moderate
Benotmane (Jul. 2021)	Moderate Due to the risk of bias	Moderate
Chavarot (Aug. 2021)	Low Due to risk of bias and inconsistency	Moderate
Del Bello (Jul. 2021)	Moderate Due to the risk of bias	Serious
Werbel (Sep. 2021)	Low Due to the risk of bias and imprecision	Moderate
Westhoff (Sep. 2021)	Low Due to the risk of bias and imprecision	Moderate

* Grade certainty meanings: very low = the true effect is probably markedly different from the estimated effect; low = the true effect might be markedly different from the estimated effect; moderate = the authors believe that the true effect is probably close to the estimated effect; high = the authors have a lot of confidence that the true effect is similar to the estimated effect. ^#^ Detailed grading of ROBINS-I can be found in the Supplementary Materials.

## Data Availability

Data are contained within the article or Supplementary Materials.

## Supplementary Materials

The following supporting information can be downloaded at: https://www.mdpi.com/article/10.3390/vaccines10010095/s1: Document S1. Systematic review search terms for PubMed (MEDLINE), EMBASE and Web of Science databases. Figure S1. A sensitivity analysis conducted using the generalized linear mixed models (GLMM) showing the rate of antibody response after the third vaccine among solid organ transplant recipients; Table S1. The risk of bias in non-randomized studies of interventions (ROBINS-I) assessment tool, including the grading of the individual eight domains.

## Abbreviations

FDA	The United States Food and Drug Administration
GLMM	Generalized linear mixed models
GRADE	Grading of Recommendations, Assessment, Development, and Evaluation
IQR	Interquartile range
MMF/MPA	Mycophenolic acid
mTOR	Mammalian target of rapamycin
PRISMA	Preferred Reporting Items for Systematic Reviews and Meta-Analysis guidelines
PROSPERO	Prospective International Register of Systematic Reviews
RCT	Randomized controlled trial
ROBINS-I	Risk of Bias in Nonrandomized Studies of Intervention
SD	Standard deviation
