# Supplementary material for: Efficacy and Safety of Third Dose of the COVID-19 Vaccine among Solid Organ Transplant Recipients: A Systemic Review and Meta-Analysis
Source: Vaccines (Basel). 2022 Jan 9;10(1):95. doi: 10.3390/vaccines10010095 (PMC8778934; doi:10.3390/vaccines10010095)

Figure S1. A sensitivity analysis conducted using the generalized linear mixed models (GLMM) showing the rate of antibody response after the third vaccine among solid organ transplant recipients.

Study	Events	Total	GLMM, Random, 95% CI
Hall 2021	33	60	0.550 [0.416; 0.679]
Masset 2021	94	136	0.691 [0.606; 0.768]
Benotmane 2021	78	159	0.491 [0.411; 0.571]
Chavarot 2021	4	62	0.065 [0.018; 0.157]
Del−Bello 2021	269	396	0.679 [0.631; 0.725]
Werbel 2021	14	30	0.467 [0.283; 0.657]
Westhoff 2021	6	10	0.600 [0.262; 0.878]
**Total (95% CI)**		**853**	**0.484 [0.299; 0.674]**

Heterogeneity: $Tau^2 = 1.0183$; $Chi^2 = 60.86$, df = 6 ($P < 0.01$); $I^2 = 90\%$

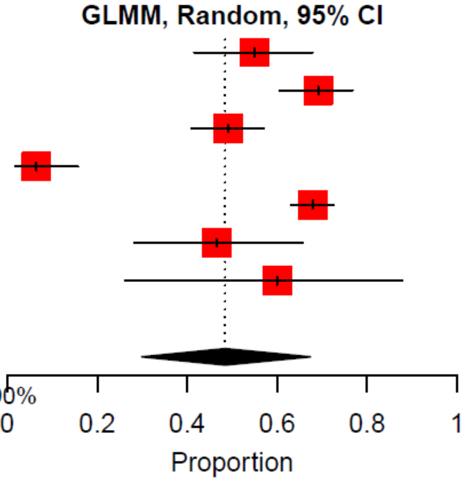

Supplement: Supplementary file 1 [file vaccines-10-00095-s001.zip › Figure S1.pdf]
