# Supplementary material for: Efficacy and Safety of Third Dose of the COVID-19 Vaccine among Solid Organ Transplant Recipients: A Systemic Review and Meta-Analysis
Source: Vaccines (Basel). 2022 Jan 9;10(1):95. doi: 10.3390/vaccines10010095 (PMC8778934; doi:10.3390/vaccines10010095)
Supplement: Supplementary file 1 [file vaccines-10-00095-s001.zip › Supplementary Online Content, Document S1.pdf]

Supplementary Online Content

Methods

Document S1 . Literature search strategy……………………………………………………………………2

Results

Table S1 ……3

Figure S1 ……4

This supplementary material has been provided by the authors to give readers additional information about the work.

Document S1. Systematic review search terms for PubMed(MEDLINE), EMBASE and Web of Science databases.

PubMed	("Three Doses" or "third dose" or "booster" or "third-dose" or "third COVID-19 vaccine" or "3rd dose" or "3 doses" or "prime-boost vaccination" or "three-dose" or "third doses" or "third shot" or "third shots")<br>AND<br>("Solid-Organ Transplant Recipients" or "immunosuppressed" or "transplant recipients" or "organ-transplant recipients")<br>AND<br>("Covid-19 Vaccine" or "messenger RNA vaccine" or "mRNA vaccine" or "Pfizer" or "mRNA-1273" or "BNT162b2" or "COVID-19 mRNA vaccine" or "SARS-CoV-2 Vaccine" or "BioNTech" or "mRNA vaccination" or "messenger RNA-based vaccines" or "anti-SARS-CoV-2")
EMBASE	('three doses' OR 'third dose' OR 'booster' OR 'third-dose' OR 'third covid-19 vaccine' OR '3rd dose' OR '3 doses' OR 'prime-boost vaccination')<br>AND<br>('solid-organ transplant recipients' OR 'immunosuppressed' OR 'transplant recipients'/exp OR 'transplant recipients' OR 'organ-transplant recipients')<br>AND<br>('covid-19 vaccine'/exp OR 'covid-19 vaccine' OR 'messenger rna vaccine'/exp OR 'messenger rna vaccine' OR 'mrna vaccine'/exp OR 'mrna vaccine' OR 'pfizer'/exp OR 'pfizer' OR 'mrna-1273'/exp OR 'mrna-1273' OR 'bnt162b2'/exp OR 'bnt162b2' OR 'covid-19 mrna vaccine' OR 'sars-cov-2 vaccine'/exp OR 'sars-cov-2 vaccine' OR 'biontech' OR 'mrna vaccination' OR 'messenger rna-based vaccines' OR 'anti-sars-cov-2')
Web of Science	('three doses' OR 'third dose' OR 'booster' OR 'third-dose' OR 'third covid-19 vaccine' OR '3rd dose' OR '3 doses' OR 'prime-boost vaccination' (Topic))<br>AND<br>('solid-organ transplant recipients' OR 'immunosuppressed' OR 'transplant recipients' OR 'transplant recipients' OR 'organ-transplant recipients' (Topic))<br>AND<br>('covid-19 vaccine' OR 'covid-19 vaccine' OR 'messenger rna vaccine' OR 'messenger rna vaccine' OR 'mrna vaccine' OR 'mrna vaccine' OR 'pfizer'/exp OR 'pfizer' OR 'mrna-1273'/exp OR 'mrna-1273' OR 'bnt162b2'/exp OR 'bnt162b2' OR 'covid-19 mrna vaccine' OR 'sars-cov-2 vaccine' OR 'sars-cov-2 vaccine' OR 'biotech' OR 'mrna vaccination' OR 'messenger rna-based vaccines' OR 'anti-sars-cov-2' (Topic))
