# Supplementary material for: Efficacy and Safety of Third Dose of the COVID-19 Vaccine among Solid Organ Transplant Recipients: A Systemic Review and Meta-Analysis
Source: Vaccines (Basel). 2022 Jan 9;10(1):95. doi: 10.3390/vaccines10010095 (PMC8778934; doi:10.3390/vaccines10010095)
Supplement: Supplementary file 1 [file vaccines-10-00095-s001.zip › Table S1.pdf]

**Table S1.** The risk of bias in non-randomized studies of interventions (ROBINS-I) assessment tool, including the grading of the individual eight domains.

| First Author (*year*) | Overall bias | Confounding | Selection of participants into the study | Classification of interventions | Deviations from intended interventions | Missing data | Measurement of outcomes | Selection of the reported result |
|---|---|---|---|---|---|---|---|---|
| Hall (*2021*)* | N/A | N/A | N/A | N/A | N/A | N/A | N/A | N/A |
| Masset (*2021*) | Moderate | Critical | Low | NI | Low | Low | Moderate | Low |
| Benotmane (*2021*) | Moderate | Critical | Low | NI | Low | Low | Low | Low |
| Chavarot (*2021*) | Moderate | Critical | Moderate | NI | Low | Low | Low | Low |
| Bello (*2021*) | Serious | Critical | Low | NI | Low | Low | Serious | Low |
| Werbel (*2021*) | Moderate | Critical | Low | NI | Low | Low | Moderate | Low |
| Westhoff (*2021*) | Moderate | Critical | Low | NI | Low | Low | Low | Low |

*Grading options include: Low, Moderate, Serous, Critical, or No Information (NI)*
*ROBINS-I is not applicable to randomized control trails.
